# Apomixis beyond trees in the Brazilian savanna: new insights from the orchid *Zygopetalum mackayi*

**DOI:** 10.1093/aobpla/plae037

**Published:** 2024-06-27

**Authors:** Gleicyanne Vieira da Costa, Mariana Ferreira Alves, Mariana Oliveira Duarte, Ana Paula Souza Caetano, Samantha Koehler, Juliana Lischka Sampaio Mayer

**Affiliations:** Departamento de Biologia Vegetal, Instituto de Biologia, Universidade Estadual de Campinas, Rua Monteiro Lobato 255, 13083-862 Campinas, SP, Brazil; Programa de Pós-Graduação em Biologia Vegetal, Instituto de Biologia, Universidade Estadual de Campinas, Rua Monteiro Lobato 255, 13083-862 Campinas, SP, Brazil; Departamento de Biologia Vegetal, Instituto de Biologia, Universidade Estadual de Campinas, Rua Monteiro Lobato 255, 13083-862 Campinas, SP, Brazil; Programa de Pós-Graduação em Biologia Vegetal, Instituto de Biologia, Universidade Estadual de Campinas, Rua Monteiro Lobato 255, 13083-862 Campinas, SP, Brazil; Instituto de Biologia, Universidade Federal de Uberlândia, Rua Ceará s/n, 38405-302 Uberlândia, MG, Brazil; Instituto de Biociências, Universidade Federal do Mato Grosso, Av. Fernando Correa da Costa 2367, 78060-900 Cuiabá, MT, Brazil; Departamento de Biologia Vegetal, Instituto de Biologia, Universidade Estadual de Campinas, Rua Monteiro Lobato 255, 13083-862 Campinas, SP, Brazil; Programa de Pós-Graduação em Biologia Vegetal, Instituto de Biologia, Universidade Estadual de Campinas, Rua Monteiro Lobato 255, 13083-862 Campinas, SP, Brazil; Departamento de Biologia Vegetal, Instituto de Biologia, Universidade Estadual de Campinas, Rua Monteiro Lobato 255, 13083-862 Campinas, SP, Brazil; Programa de Pós-Graduação em Biologia Vegetal, Instituto de Biologia, Universidade Estadual de Campinas, Rua Monteiro Lobato 255, 13083-862 Campinas, SP, Brazil

**Keywords:** adventitious embryony, asexual reproduction, Cerrado, embryology, geographical parthenogenesis, megagametogenesis, megasporogenesis, Orchidaceae, polyploidy, polyembryony

## Abstract

n the Neotropics, the focus of apomictic studies predominantly centres on trees within the Brazilian savanna, characterized, mostly as sporophytic and facultative, associated with polyploidy and polyembryony. To enhance our understanding of the mechanisms governing apomixis and sexual reproduction in tropical herbaceous plants, we clarify the relationship between apomixis, chromosome counts, and polyembryony in the epiphytic orchid *Zygopetalum mackayi*, which forms a polyploid complex within rocky outcrops in both the Brazilian savanna and the Atlantic forest. To define embryo origins and describe megasporogenesis and megagametogenesis, we performed manual self-pollinations in first-day flowers of cultivated plants, considering all three cytotypes (2*x*, 3*x*, 4*x*) of this species. Flowers and fruits at different stages were collected to describe the development and morphology of ovules and seeds considering sexual and apomictic processes. As self-pollination treatments resulted in high fruit abortion in diploids, we also examined pollen tube development in aborted flowers and fruits to search for putative anomalies. Megasporogenesis and megagametogenesis occur regularly in all cytotypes. Apomixis is facultative and sporophytic, and associated with polyploid cytotypes, while diploid individuals exclusively engage in sexual reproduction. Polyembryony is caused mainly by the production of adventitious embryos from nucellar cells of triploids and tetraploids, but also by the development of multiple archesporia in all cytotypes. Like other apomictic angiosperms within the Brazilian savanna, our findings demonstrate that apomixis in *Z. mackayi* relies on pollinators for seed production. We also consider the ecological implications of these apomictic patterns in *Z. mackayi* within the context of habitat loss and its dependence on pollinators.

## Teaser

Our study on the leaf-litter orchid *Zygopetalum mackayi* , found in Brazilian high-elevation rocky complexes, reveals crucial insights into its reproductive mechanisms. We found that apomixis in this orchid is facultative and sporophytic and linked to polyploid cytotypes. Polyembryony arises mainly from adventitious embryos in polyploids but also from multiple archesporia, a rare phenomenon in orchids. Apomictic seed formation depends on pollination, highlighting an ecological vulnerability due to declining bee species populations. These findings align with patterns seen in other apomictic plants from the Brazilian savanna, highlighting the interconnected roles of polyploidy, pollination, and environmental factors in the reproductive strategies of *Z. mackayi* .

## Introduction

The elucidation of the origins, maintenance, and diversification of asexual lineages constitutes a pivotal debate in the field of evolutionary biology (Hojsgaard *et al.* 2014). Apomixis, the production of seeds asexually, is known for more than 1400 species, being phylogenetically widespread within the angiosperms (Hojsgaard and Pullaiah 2022). Apomictic embryos can arise through three distinct developmental mechanisms (Koltunow 1993). Sporophytic apomixis, also known as adventitious embryony, involves the direct formation of adventitious embryos from somatic cells within the ovule. In contrast, gametophytic apomixis entails embryo development via egg cell parthenogenesis within an unreduced embryo sac. This unreduced embryo sac may derive from either a megaspore mother cell (diplospory) or a nucellar cell (apospory). Commonly, polyembryonic seeds, containing both sexual and asexual embryos, can be associated with both sporophytic and aposporous apomixis (Naumova 1993).

Most knowledge about the origin and diversification of apomictic plant lineages comes from gametophytic apomixis from northern latitudes (Hojsgaard and Hörandl 2019). Compared to their sexual relatives, apomictic plants in the northern hemisphere are typically polyploid, possess broader distributions, thrive at higher altitudes, and inhabit regions formerly covered by glaciers (Hojsgaard *et al.* 2014). However, the link between apomixis and biogeographic traits in tropical species remains relatively understudied. Available studies suggests that apomixis in neotropical plants is primarily sporophytic, facultative, and associated to polyploidy and polyembryony (e.g. Mendes-Rodrigues and Oliveira 2012; Sampaio *et al.* 2013; Alves *et al.* 2016; Caetano and Oliveira 2022; Marinho *et al.* 2023). Despite the recognition of apomixis in plants for over a century (Hojsgaard and Pullaiah 2022), our understanding of apomictic tropical plants remains incomplete and it is clearly underestimated, as demonstrated by recent studies in the Andes (Ptáček *et al.* 2024) and for tropical families, such as Podostemaceae (Silva-Batista *et al.* 2020) and Melastomataceae (Caetano and Oliveira 2022). In this context, apomictic tropical species from highly diverse plant groups present promising new models for investigating apomixis in the Neotropics.

Orchids comprise one of the most diverse lineages of angiosperms with diverse apomictic systems. Out of 905 accepted orchid genera (WFO 2022), apomictic species are known for 39 species and 16 genera (Xiao *et al.* 2021; Hojsgaard and Pullaiah 2022). Among species with available embryological studies, three are diplosporic and 28 exhibit adventitious embryony. Four of these depend on pollination for seed development, and 27 are autonomous (Xiao *et al.* 2021; Hojsgaard and Pullaiah 2022). At first, the species *Zygopetalum mackayi* Hook. is the only orchid to combine apospory and sporophytic apomixis (Suessenguth 1923). Over a century ago, Hurst (1897) described apomictic reproduction of this species, a leaf-litter orchid found in high-elevation rocky complexes within the Brazilian savanna and the Atlantic forest (Gomes *et al.* 2018). Suessenguth (1923) further described the simultaneous occurrence of gametophytic and sporophytic apomixis, the production of polyembryonic seeds, and the occurrence of polyploids in this species. He also suggested the absence of sexual embryos and that the pollen tube was only necessary to stimulate the development of apomictic embryos. Later, Afzelius (1959) proposes the occurrence of obligate apomixis in *Z. mackayi* but reported embryo formation strictly through sporophytic apomixis. However, both studies were based on one or two specimens and only conducted self-pollinations or cross-pollinations with phylogenetically very distinct species (*Calanthe* and *Coelogyne*). Later, Gomes *et al.* (2018) showed *Z. mackayi* exhibits three different cytotypes (diploid, 2*n* = 48; triploid, 2*n* = 72; and tetraploid, 2*n* = 96). Diploids and tetraploids are geographically structured and associated with different climatic conditions, while triploids are F_1_ hybrids found in a contact zone where diploids and tetraploids coexist (Gomes *et al.* 2018; Moura *et al.* 2020). Additionally, studies of reproductive biology (Campacci *et al.* 2017) and population genetics (Moura *et al.* 2020) suggested *Z. mackayi* primarily reproduces sexually and facultatively by apomixis (as a result of either self- or cross-pollinations), with pollination-dependent embryo formation for fruit and seed development (Campacci *et al.* 2017).

Despite the wealth of information regarding cytogenetics, genetics, reproductive biology, and ecological niche modelling of *Z. mackayi* (Campacci *et al.* 2017; Gomes *et al.* 2018; Moura *et al.* 2020), previous studies have been inconsistent about the developmental mechanism of apomixis (Suessenguth 1923; Afzelius 1959). In order to deepen our understanding of apomixis in tropical plants, we here clarify the relationship among apomixis, chromosome numbers and polyembryony in this species. Specifically, we seek to address the following questions: (1) Is apomixis facultative and co-occurring with sexual reproduction? (2) What is the origin of apomictic embryos? (3) Is apomixis associated with polyploidy? (4) Is apomixis associated with polyembryony? Results are discussed in the context of apomictic patterns in the Brazilian savanna.

## Material and Methods

### Study system and sampling

*Zygopetalum mackayi* is found in high-altitude rocky complexes in southeastern Brazil, situated within the Brazilian savanna and the Atlantic forest. This taxon is part of a heteroploid agamic complex that comprises up to 11 distinct species, which have never been subject to a taxonomic revision (Hoehne 1953; Pupulin 2009). Given the clear disjunct distribution of *Z. maculatum* (Kunth) Garay, limited to Bolivia and Peru (S. Koehler, unpublished data), we have adopted a species concept that places emphasis on the genetic and ecological coherence among populations in southeastern Brazil, which we now recognize as *Z. mackayi* (Campacci *et al.* 2017; Moura *et al.* 2020). *Zygopetalum mackayi* primarily flowers during the dry season, from April to July, with a secondary, less intense flowering peak between December and January (Campacci *et al.* 2017; Nunes *et al.* 2017). Flowers are 4–6 cm wide, flabellate. Fully developed fruits are 6–8 cm in length, 2 cm wide, and contain thousands of seeds about 2 mm in length.

Plants used in this study were cultivated for a minimum of three years in the orchid nursery of Universidade Estadual de Campinas (São Paulo, Brazil). Vouchers were deposited at UEC. A total of 73 specimens from 17 distinct localities were subjected to artificial self-pollination treatments to estimate fruit set rates and to elucidate the embryo origins while describing megasporogenesis, megagametogenesis, and seed development (see Supporting Information—Table S1). Ploidy was determined by flow cytometry and confirmed by chromosome counts by Gomes *et al.* (2018). We conducted a chi-square test for residuals to compare the impact of ploidy on fruit set, with a significance level of 0.05 (**see Supporting Information—**Table S2). The analyses were carried out using SPSS Statistics for Windows v. 29.0.1.0 (IBM Corp., Armonk, USA).

### Anatomical studies

In order to determine the origins of sexual and asexual embryos and describe megasporogenesis and megagametogenesis, we conducted manual self-pollinations on first-day flowers, considering 26 flowers from diploids individuals (*n *= 17 specimens), 27 flowers from triploids individuals (*n *= 6), and 60 flowers from tetraploids individuals (*n *= 32). We standardized pollination treatments and performed only self-pollinations based on a previous reproductive biology study of *Z. mackayi*, which gathered evidence that pollen origin (i.e. treatments of self-pollinations and intrapopulation or between populations cross-pollinations) affects the viability and number of embryos per seed (Campacci *et al.* 2017). For our anatomical observations, we considered the definition of fruit as a structure developing from the gynoecium of one flower as the result of pollination or parthenocarpy (Pax 1890; Bobrov and Romanov 2019). Flowers and fruits were collected at irregular intervals ranging from 1 to 134 days after pollination (DAP). The samples were fixed by immersion in a solution of 4% formaldehyde, and 2.5% glutaraldehyde in 0.05 M phosphate sodium buffer (modified from Karnovsky 1965). Subsequently, they were gradually dehydrated in an ethanol series before being embedded in resin (Leica Historesin®). Sections of 3–5 μm thick were obtained with a manual rotary microtome (Leica®), stained with 0.05% Toluidine Blue 0.05% in citrate buffer, pH 4.5 (Sakai 1973), and mounted with synthetic resin Entellan (Merck^®^). The analyses were carried out using an Olympus BX51 optical microscope equipped with a digital camera Olympus DP71.

### Pollen tube development

To analyse pollen tube development, we conducted self-pollinations on diploids and tetraploids individuals, considering nine flowers from six specimens of each cytotype. We collected the distal portion of the gynoecium and/or the median part of the fruit 1–134 days after pollination (Table 1). For anatomical studies, samples were fixed in ethanol 70% for 48 h, then softened in a NaOH 10N solution at 60°C for 15 min, and subsequently washed in distilled water and left overnight in a 1% aniline blue solution in a potassium phosphate buffer, pH 7.0 (modified from Martin 1959). Analyses and photomicrographs were conducted using an Olympus BX51 optical microscope equipped with epifluorescence and captured with a digital camera Olympus DP71.

**Table 1.** Developmental stages of ovule and embryos of flowers and fruits of diploids, triploids and tetraploids *Zygopetalum mackayi*. Time intervals are indicated as days after pollination. AEPs = adventitious embryo precursor cells.

Developmental stage	Diploid	Triploid	Tetraploid
Placental proliferation and ovule initiation differentiation	1–12	1–12	1–12
Pollen tubes arrive in ovary	13	13	13
Archesporial cell differentiation	20	20	20
Megaspore mother cell differentiation	22	30	22
Meiosis and megaspore dyad/tetrad formation	24	38	24
Degeneration of the three micropylar megaspores; differentiation of AEPs in the nucleus of polyploids	24	40	32
Mature embryo sac	60	45	40
Egg cell fertilization	64	60	42
Zygote and endosperm formation	66	60	44
Endosperm degeneration	70	64	46
First divisions of the zygote and AEPs in polyploids; young globular embryos and suspensor differentiation	70	66	48
Seed coat tegmen begin to degenerate; seed testa expand and lignify; differentiation of protoderm in the embryo	82	78	66
Mature embryos and interruption of their mitotic activity; mature seeds	102	90	86
Seed coat with single thin layer (testa); suspensor degeneration	134	98	100

## Results

**Figure 1.**
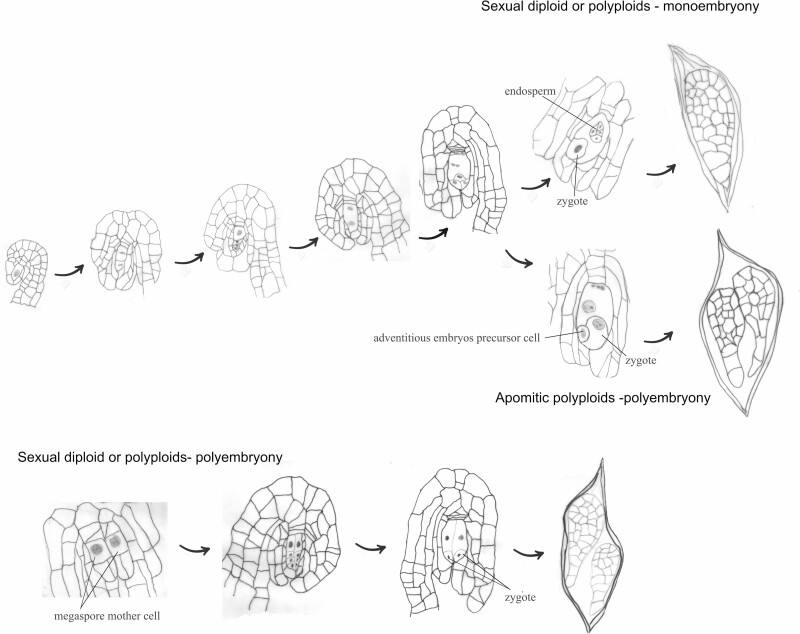
Schematic model showing the different reproductive processes in diploid and polyploid individuals of *Zygopetalum mackayi*.

Fruits and seeds were formed after hand pollination experiments. Sexual and asexual embryo development are summarized in Fig. 1.

### Sexual development of ovules and seeds

**Figure 2.**
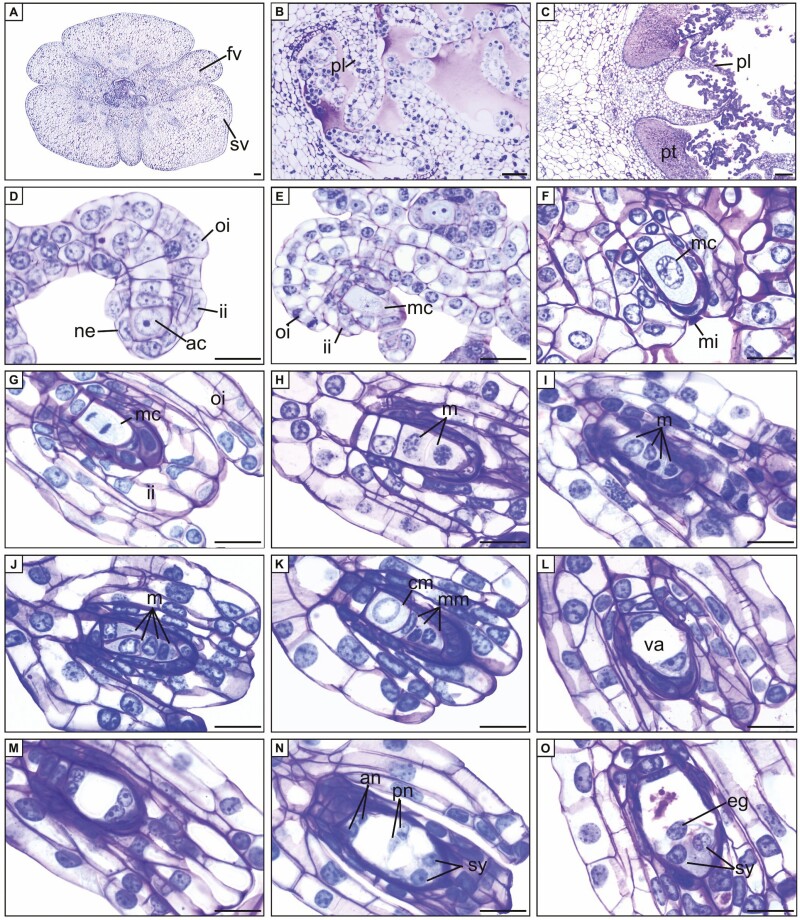
Longitudinal sections of ovules of *Zygopetalum mackayi*. (A) Overview of the ovary of the anthetic flower. (B) Placenta with ovules at the beginning of development. (C) Pollen tube at placenta 20 days after pollination. (D) Differentiation of the initial archesporial cell and formation of the integuments. (E) Megaspore mother cell. (F) Megaspore mother cell and integuments. (G) Megaspore mother cell during meiosis I. (H) Megaspore dyad. (I) Linear tetrad of megaspores. (J) Inverted T-shaped tetrad of megaspores. (K) Chalazal megaspore expanding and degenerating micropylar megaspores. (L) Binucleate megagametophyte. (M) Tetranucleate megagametophyte. (N) Mature megagametophyte. (O) Synergids and egg cell. ac = archesporial cell; an = antipodes; cm = chalazal megaspore; eg = egg cell; fv = fertile valve; ii = inner integument; m = megaspore; mc = megaspore mother cell; mi = micropyle; mm = micropylar megaspores; ne = nucellar epidermis; oi = outer integument; pl = placenta; pn = polar nuclei; pt = pollen tube; sv = sterile valve; sy = synergids; va = vacuole. A, B, E, G, I, J, L, M, O = tetraploid cytotype; D, H = triploid cytotype; C, F, K, N = diploid cytotype. Scale bars: A-C = 20 μm; D-O = 10 μm.

All cytotypes exhibited similar patterns of ovule and seed sexual development. The ovary consisted of three carpels divided into six valves: three fertile placental regions and three sterile valves (Fig. 2A). In the anthetic flower, the fertile valves only contained primordia ovules (Fig. 2B). Following manual self-pollination, the pollen grains germinated and the pollen tubes grew along the stylar canal while the placenta proliferated through intense mitotic activity (Fig. 2C). The pollen tubes reached the base of the fruit at about 13 DAP and remained in the placental region until the ovule matured (Fig. 2C). Ovule development begun around 20 DAP (Table 1). Ovule differentiation was not synchronous within the same fruit or among cytotypes (Table 1).

In each ovule primordium, a cell from the subepidermal layer underwent differentiation, forming an archesporial cell with a clearly visible nucleus (Fig. 2D). During this developmental phase, periclinal cell divisions took place in the nucellar epidermis, resulting in the formation of both the outer and inner integuments (Fig. 2D and E). The archesporial cell increased in volume and did not divide, directly giving rise to the megaspore mother cell (MC), characterizing the ovule as tenuinucellate (Fig. 2E and F). The initial phase of meiotic division in the MC yielded a megaspore dyad (Fig. 2G and H) while the subsequent phase of meiotic division resulted in a tetrad of megaspores (Fig. 2I and J). At this stage, the inner integument had elongated, and its margins completely enclosed the nucellar epidermis, delimiting the micropyle (Fig. 2G–J). The chalazal megaspore became functional, while the three micropylar megaspores degenerated (Fig. 2K).

The functional megaspore increased in volume, initiating the first mitotic cycle and giving rise to a binucleate megagametophyte. Subsequently, a large central vacuole formed, with each nucleus relocating to one pole of the megagametophyte (Fig. 2L). Simultaneously, both nuclei underwent the second mitotic cycle, resulting in a tetranucleate megagametophyte (Fig. 2M). This was followed by the third and final mitotic cycle, culminating in the formation of an octanucleate megagametophyte. During cellularization, one nucleus from the chalazal pole and one from the micropylar pole migrated towards the centre of the megagametophyte, constituting the polar nuclei of the central cell (Fig. 2N). The remaining nuclei at the micropylar pole organized into two synergids and an egg cell, forming a triangular arrangement (Fig. 2O), while the three remaining nuclei at the chalazal pole develop into the antipodals (Fig. 2N). These patterns were indicative of a *Polygonum*-type embryo sac development (monosporic, eight-nucleate, and seven-celled). At this stage, the ovules were anatropous and bitegmic.

**Figure 3.**
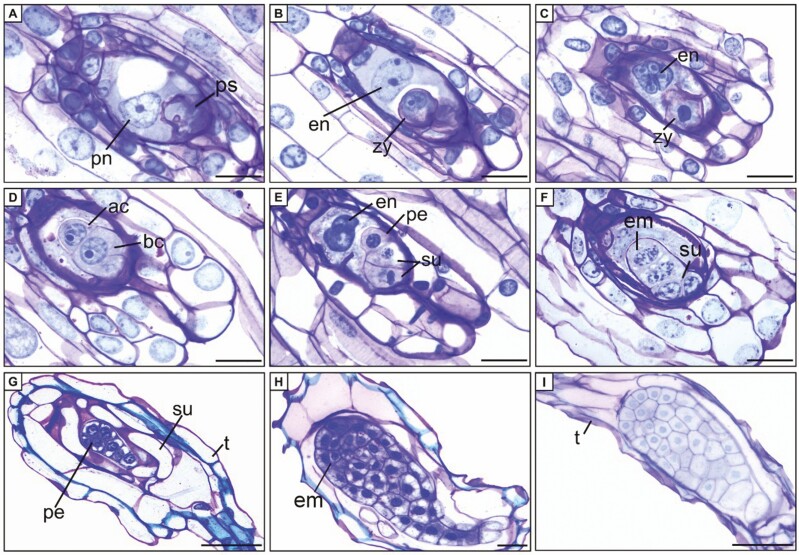
Longitudinal sections of ovules and seeds of *Zygopetalum mackayi.* (A) Penetrated synergid. (B) Zygote and primary endosperm nucleus. (C) Zygote and nuclear endosperm. (D) Embryo with two cells. (E–G) Embryo and suspensor. (H–I) Mature seed. ac = apical cell; bc = basal cell; em = embryo; en = endosperm; pe = pro-embryo; pn = polar nuclei; ps = penetrated synergid; t = testa (seed coat); su = suspensor; zy = zygote. A, B, D, F, H = tetraploid cytotype; E = triploid cytotype; C, G, I = diploid cytotype. Scale bars: A–F = 10 μm; G–I = 20 μm.

Fertilization took place between 42 and 64 DAP, and it was identified by the dense cytoplasmic staining of the synergid. Zygote organization occurred between 44 and 66 DAP (Fig. 3A–C; Table 1). The polar nuclei fused with the male gamete nucleus, forming the primary endosperm nucleus, which then underwent division to produce up to four nuclei, all of which further degenerate (Fig. 3C). The initial mitotic division of the zygote was asymmetric, resulting in a smaller apical cell and a larger basal cell (Fig. 3D). The apical cell gave rise to the embryo, while the basal cell forms the suspensor (Fig. 3E–G). The apical cell underwent further divisions without a defined pattern, leading to the formation of the pro-embryo. Simultaneously, the suspensor cells became elongated and vacuolated, occupying a significant portion of the seed (Fig. 3E–G).

Throughout embryogenesis, the cells of the inner integument degenerated, while the outer integument elongated to develop into the seed coat (testa) (Fig. 3G–I). As the embryo grows, the suspensor becomes compressed and eventually degenerates. The mature seed exhibits a transparent testa, formed by a single layer of lining cells, which serves to protect the globular embryo (Fig. 3I). In mature seeds, there was no differentiation of meristems or cotyledons in the embryo (Fig. 3I).

**Figure 4.**
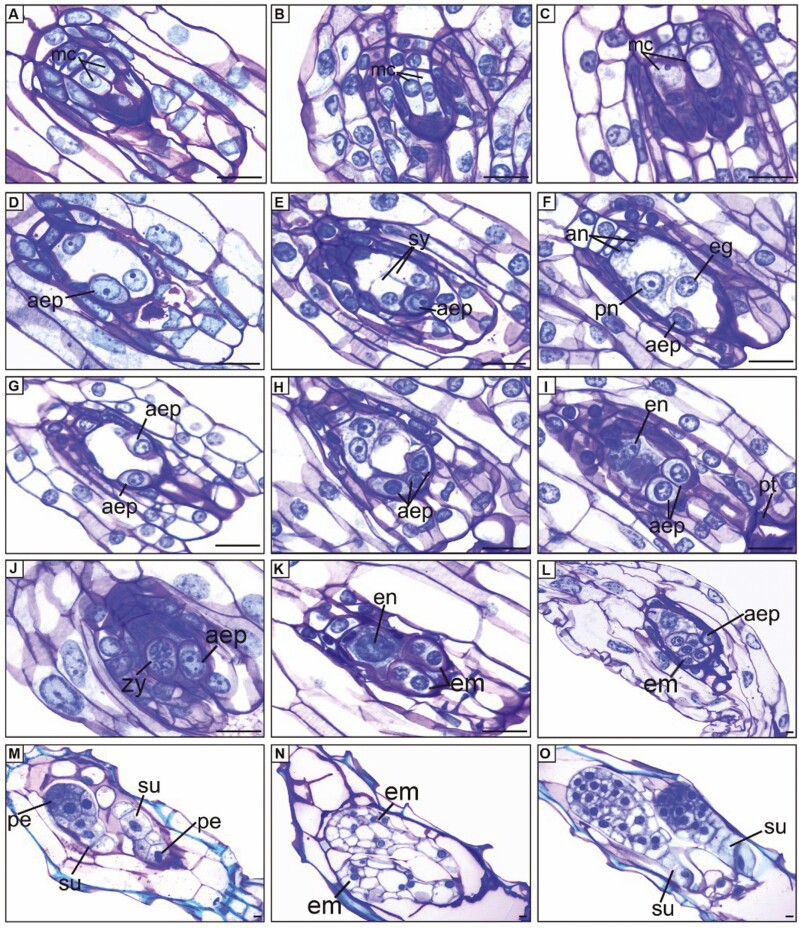
Longitudinal sections of ovules and young seeds in *Zygopetalum mackayi.* (A–C) Differentiation of two megaspore mother cell in the same ovule. (D–E) Adventitious embryo precursor cell (AEP) in the integument of the micropylar region. (F) AEP and egg cell. (G) Two AEPs in the micropylar region. (H) Three AEPs in the micropylar region. (I) AEPs and endosperm. (J) Zygote and AEP. (K) Two zygotes and endosperm. (L) Five cell embryo and AEP in the micropylar region. (M-O) Sexual and adventitious embryo. aep = adventitious embryos precursor cells; an = antipodes; eg = egg cell; em = embryo; en = endosperm; mc = megaspore mother cell; pe = pro-embryo; pn = polar nuclei; pt = pollen tube; su = suspensor; sy = synergids, zy = zygote. C, D, F, H, I, K-O = tetraploid cytotypes; G, J = triploid cytotype; A, B = diploid cytotype. Scale bars: A-I = 10 μm, J–O = 20 μm.

We also observed the differentiation of two MCs in the same ovule (i.e. multiple archesporia): three in diploids (11%), eight in triploids (29%), and none in tetraploids (Fig. 4A-C). When this phenomenon occurred, the MCs underwent meiosis and formed two tetrads of megaspores, with chalazal megaspores being functional (Fig. 4A–C). Subsequently, the megaspores underwent three mitotic cycles, followed by cellularization, resulting in the development of two reduced megagametophytes within the same ovule.

### Sporophytic apomixis originating supernumerary embryos in polyploids

In triploid and tetraploid cytotypes, we observed the differentiation of nucellar cells in the micropylar region of the megagametophyte 32–40 DAP (Fig. 4D–G). These cells increased in volume, invaded the interior of the megagametophyte, and were identified as adventitious embryos precursor cells (AEPs). We observed the differentiation of up to three AEPs in the same ovule (Fig. 4H). AEPs could be distinguished from the egg cell by their peripheral and lateral position in the megagametophyte, and by their dense cytoplasm, central nucleus, and thickened cell wall. AEPs remained unchanged until the pollen tube penetrated the synergid, leading to double fertilization, up to 42–64 DAP (Fig. 4F–I). Their development in adventitious embryos occurred simultaneously with the sexual embryo or after the initiation of the sexual embryo development (Fig. 4J–L). Distinguishing between adventitious and sexual embryos was challenging because they were structurally similar, and the adventitious embryos were situated in the micropylar region (Fig. 3K; 4M–O). However, while the sexual embryo’s suspensor was located in the micropylar region of the seed (Fig. 4M), the suspensor of the adventitious embryo was observed in both the micropylar and chalazal regions (Fig. 4M–O).

### Fruit set and pollen tube development

**Figure 5.**
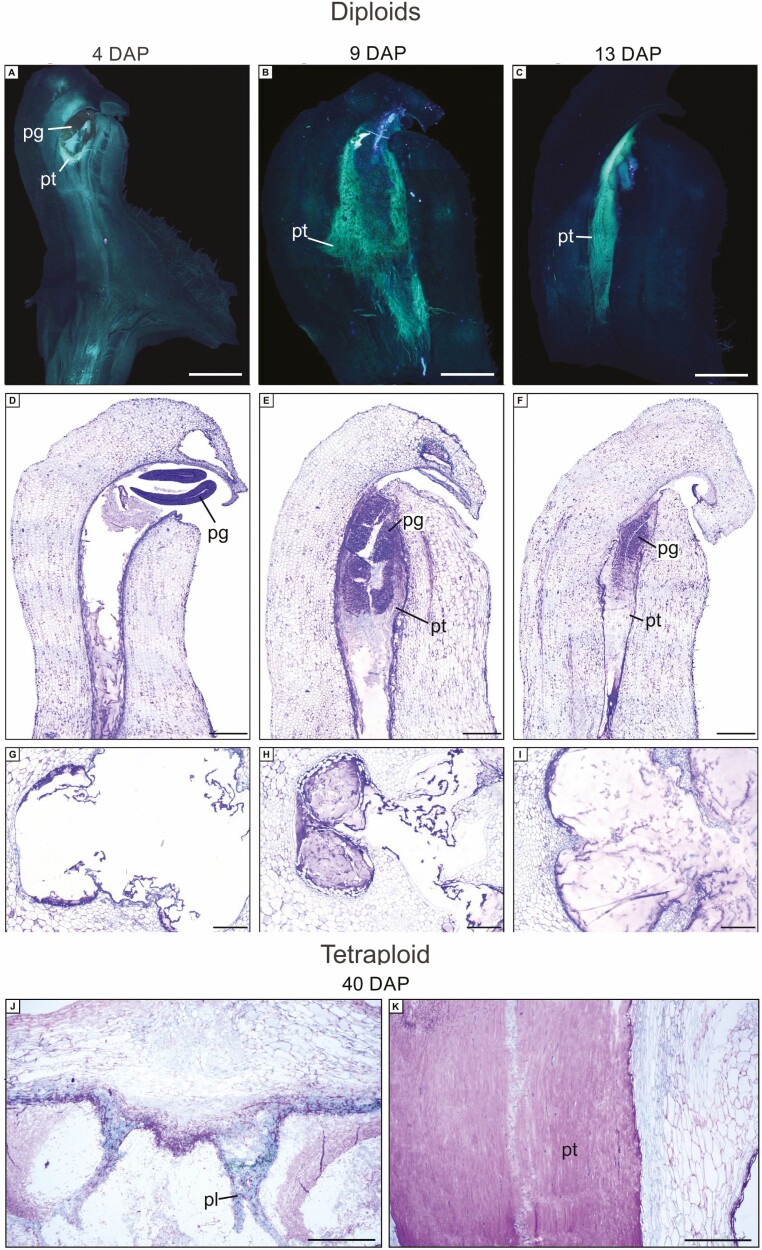
Longitudinal section of column of *Zygopetalum mackayi*, showing pollen tube growth after self-pollination. (A–C) epifluorescence microscope image; (D–K) light microscopy image. (A, D) Pollen grain germination four days after pollination. (B, E) Growth of pollen tubes in column nine days after pollination. (C, F) Pollen tubes in ovary 13 days after pollination. (G) Degenerated placenta nine days after pollination. (H) Degenerated placenta 13 days after pollination. (I) Degenerated placenta 40 days after pollination. (J) Degenerated placenta 40 days after pollination. (K) Pollen tubes growth in aborted fruits 40 days after pollination. pg = pollen grain; pl = placenta; pt = pollen tube. A–I = diploid cytotype; J–K = tetraploid cytotype. Scale bars: A–F = 200 μm; G–I = 100 μm.

Fruit set from self-pollinations in diploids resulted in a lower fruit set (34.4%) when compared to triploids (59.3%) and tetraploids (68.5%) (*X*^2^ = 20.43, df = 2, *P* > 0.001, Table 2, see Supporting Information**—**Table S2). We did not observe any interruption in pollen tube growth or irregular callose deposition in aborted flowers of each cytotype (Fig. 5A–K). In diploids and tetraploids (triploids not observed), pollen grains germinated on the stigma, and pollen tube developed into the style 3-4 DAP (Fig. 4A and D). After nine DAP, pollen tubes covered almost the entire style (Fig. 5B and E), and by 13 DAP, pollen tubes reached the base of the gynostemium and entered the fruit locule (Fig. 5C and F). Pollen tubes reached the fruit locule in aborted fruits of diploids (Fig. 5A–C) and tetraploids (Fig. 5J and K). Moreover, we did not observe placental differentiation and ovule formation in diploid aborted fruits (Fig. 5G–I). Placenta and ovule primordia initiated development in tetraploid aborted fruits but ceased and degenerated (Fig. 5J).

**Table 2.** Percentage of fruit set from self-pollination treatments in *Zygopetalum mackayi*. Sample size/number of fruits developed to completion is indicated.

Cytotype	Sample size	Fruits developed to completion
Diploid	64	22 (34.4%)
Triploid	27	16 (59.3%)
Tetraploid	130	89 (63.8%)

## Discussion

In this study, we analysed fruit development of individual cytotypes and provided developmental details of sporophytic apomixis in *Z. mackayi*, since we identified differentiation of nucellar cells into AEPs in triploids and tetraploids. Moreover megasporogenesis, megagametogenesis and the double fertilization occurred regularly in all cytotypes of *Z. mackayi*, which excludes the occurrence of gametophytic and/or obligate apomixis in this species. Apomixis in *Z. mackayi* is, therefore, *facultative* and *sporophytic*, and associated with the polyploid cytotypes, with no apomictic reproduction observed in diploid individuals. Effective sexual events in all cytotypes was confirmed by pollen tubes remains in the micropylar region of penetrated synergids, zygote formation, and polar nuclei fertilization, indicators of sexual events.

We also showed polyembryony in *Z. mackayi* is caused by two different processes. Besides the production of adventitious embryos from nucellar cells of triploids and tetraploids, polyembryonic seeds may also result from multiple archesporia in diploids and triploids cytotypes. The occurrence of multiple archesporia is a pre-meiotic event that originates megaspore mother cells, which will develop sister tetrads followed by two independent embryo sacs within a single ovule (e.g. Carmo-Oliveira *et al.* 2020). This is a rare event in the Orchidaceae, so far reported only for *Lecanorchis japonica* (Johri *et al.* 1992). Thus, although the production of adventitious embryos in *Z. mackayi* is more common than the occurrence of multiple archesporial cells, polyembryony should not be used as direct evidence of apomixis in this species. Moreover, it is possible that monoembryonic seeds bear a single apomictic embryo instead of a sexual one, as it is not possible to distinguish between them when fully matured.

Apomixis in *Z. mackayi* is also dependent on pollination, as only pollen tube growth trigger ovule development in all cytotypes, as previously indicated by Campacci *et al.* (2017). The development of apomictic embryos begins after the sexual ones but adventitious embryo precursor cells appeared much earlier than sexual fertilization. Male sexual function is generally conserved in apomictic orchids, not for endosperm development, but because pollination is necessary for ovule development (Zhang and O’Neill 1993). In fact, according to Hojsgaard and Pullaiah (2022), most confirmed apomictic orchids (55%) develop endosperm autonomously, i.e. without fertilization of polar nuclei (see also Xiao *et al.* 2021). This contrasts with most other apomictic angiosperms (Johri 1992), in which pollination followed by fertilization of polar nuclei and endosperm formation is required for apomictic embryo development (pseudogamy sensu Nogler 1984). Although a high number of apomictic angiosperms also develop endosperm autonomously (Hojsgaard and Pullaiah 2022), the endosperm in orchid seeds is non-functional, as it is never formed or degenerates after polar nuclei fertilization (Yeung 2017).

The occurrence of apomixis in orchids exhibits a pronounced bias towards terrestrial species of the subfamily Orchidoideae. Among the 30 confirmed apomictic orchid species, merely four (13%), among them *Z. mackayi*, belong to subfamily Epidendroideae (a mostly epiphytic clade), which encompasses over four times the number of species compared to the subfamily Orchidoideae (a mostly terrestrial clade) (Atwood 1986). Within Epidendroideae apomictic orchids, two species, *Mormolyca cleistogama* (as *Maxillaria cleistogama*) and *Epidendrum nocturnum* are epiphytic (Illg 1977; Veyret 1982). The other two species, *Z. mackayi* and *Cepridium acuminatum* (as *Malaxis walchitii*) (Sood and Ram 1995), primarily exhibits a terrestrial habit in well-drained substrates, being occasionally observed as epiphytes. Ovules in terrestrial orchid species are more advanced in development at the time of pollination compared to their epiphytic counterparts (Mayer *et al.* 2021). This may explain the prevalence of apomixis in terrestrial species of the subfamily Orchidoideae.

Apomixis may be a result of different factors, including gene expression. Barcaccia *et al.* (2020) showed apomixis independently evolved several times in angiosperms, presenting different developmental pathways that are controlled by distinct genetic factors. Several candidate genes have been reported for polyembryony and nucellar embryo formation in other angiosperm families (e.g. Aron *et al.* 1998; Shimada *et al.* 2018; Fei *et al.* 2021). For the orchid *Cymbidium sinense*, there are drastic differential expression patterns in intermediate to later periods of sexual ovule development (Zeng *et al.* 2022). Therefore, it is possible that the less advanced state of ovules in epiphytic orchids at the time of pollination may also limit the occurrence of apomixis because genes related to the development of adventitious embryos are not active. Dependence on pollination for ovule development may be an additional, yet unexplored, constraint determining the lack of more autonomous apomixis in orchids, an even rarer phenomenon in the family, known for *Cynorkis* spp. (Veyret 1972); *Genoplesium apostasioides* (Sorensen *et al.* 2009), *Habenaria malintana* (Zhang and Gao 2018), *Rhomboda tokioi* (Xiao *et al.* 2021), *Spiranthes cernua* (Catling 1982; Schmidt and Antlfinger 1992), and *Zeuxine strateumatica* (Seshagiriah 1941). Comparative studies on gene expression in apomictic (both autonomous and pollination dependent) and sexually reproducing orchids, especially comparing species from Orchidoideae and Epidendroideae subfamilies, are needed to fully understand the extent of apomixis in this family and the molecular and developmental factors influencing it.

Our results on apomixis in *Z. mackayi* agree with those previously obtained for other apomictic plants from the Brazilian savanna, which also depend on pollinators for embryo formation. Studies with trees from the Brazilian savanna reported facultative sporophytic apomixis associated with polyploidy, polyembryony, and pseudogamy in *Eriotheca* spp. (Malvaceae), *Handroanthus* spp., *Anemopaegma* spp. (Bignoniaceae), *Inga laurina* (Fabaceae) (Sampaio *et al.* 2013; Alves *et al.* 2016; Mendes *et al.* 2018; Mendes-Rodrigues *et al.* 2019). The fact that even asexual reproduction in *Z. mackayi* requires pollination may have significant ecological implications. The species is pollinated by bees of the genus *Bombus* (Campacci *et al.* 2017), whose populations are declining worldwide (Cameron and Sadd 2020). Therefore, in addition to the rapid habitat loss in the Brazilian savanna due to agricultural expansion (Bonanomi *et al.* 2019), the dependence on pollinators that are becoming rarer may compromise population growth in *Z. mackayi*, both through sexual and asexual reproduction. In conclusion, this study shed light on the mechanisms governing apomixis and sexual reproduction in *Z. mackayi*. Our findings show that apomixis in this species is facultative and sporophytic, primarily associated with polyploid cytotypes, while diploid individuals exclusively engage in sexual reproduction. Moreover, we have elucidated the processes underlying polyembryony, emphasizing the importance of distinguishing between adventitious embryos and those resulting from multiple archesporia. Finally, like most other apomictic angiosperms from the Brazilian savanna, we corroborated that apomixis in *Z. mackayi* is dependent on pollination services for seed formation Campacci *et al.* (2017), which sustainable use will require conservation policies.

## Supporting Information

The following additional information is available in the online version of this article –

Table S1. Outcome of hand-pollination treatments for each flower sampled in diploid, 2*n* = 48 (2x), triploid, 2*n* = 72  (3x), and tetraploid, 2*n* = 96 (4x) specimens of *Zygopetalum mackayi*. (0) Fruit abortion, (1) Fruits developed to completion. Ploidy, locality, and specimen ID are indicated.

Table S2. Chi-square analyses test for residuals to compare the impact of ploidy on fruit set for each cytotype of *Zygopetalum mackayi*. Diploid, 2*n* = 48 (2x); triploid, 2*n* = 72 (3x); and tetraploid, 2*n* = 96 (4x). Significance level of 0.05.

plae037_suppl_Supplementary_Tables

## Data Availability

All data supporting the findings of this study are available as supporting information.
