# Supplementary material for: Apomixis beyond trees in the Brazilian savanna: new insights from the orchid Zygopetalum mackayi
Source: AoB Plants. 2024 Jun 27;16(4):plae037. doi: 10.1093/aobpla/plae037 (PMC11237986; doi:10.1093/aobpla/plae037)
Supplement: plae037_suppl_Supplementary_Tables [file plae037_suppl_supplementary_tables.pdf]

Table S1. Outcome of hand-pollination treatments for each flower sampled in diploid (2x), triploid (3x) and tetraploid (4x) specimens of *Zygopetalum mackayi*. (0) Fruit abortion, (1) Fruits developed to completion. Ploidy, locality and specimen ID are indicated.

**Ploidy**	**Locality**	**Specimen ID**	**Outcome**
2X	Cotia/SP	ZM029	0
2X	Cotia/SP	ZM029	0
2X	Cotia/SP	ZM029	0
2X	Cotia/SP	ZM029	1
2X	Cotia/SP	ZM030	0
2X	Cotia/SP	ZM030	0
2X	Cotia/SP	ZM030	0
2X	Cotia/SP	ZM030	1
2X	Cotia/SP	ZM035	0
2X	Cotia/SP	ZM035	0
2X	Cotia/SP	ZM035	0
2X	Cotia/SP	ZM035	0
2X	Santo Antônio do Itambé/MG	ZM176	1
2X	Santo Antônio do Itambé/MG	ZM176	1
2X	Santo Antônio do Itambé/MG	ZM186	0
2X	Santo Antônio do Itambé/MG	ZM186	0
2X	Santo Antônio do Itambé/MG	ZM186	0
2X	Santo Antônio do Itambé/MG	ZM186	0
2X	Santo Antônio do Itambé/MG	ZM186	0
2X	Santo Antônio do Itambé/MG	ZM186	0
2X	Santo Antônio do Itambé/MG	ZM186	0
2X	Santo Antônio do Itambé/MG	ZM186	0
2X	Santo Antônio do Itambé/MG	ZM186	1
2X	Alvarenga/MG	ZM351	0
2X	Alvarenga/MG	ZM351	0
2X	Alvarenga/MG	ZM351	0
2X	Alvarenga/MG	ZM364	1
2X	Alvarenga/MG	ZM364	1
2X	Alvarenga/MG	ZM369	0
2X	Alvarenga/MG	ZM369	0
2X	Alvarenga/MG	ZM369	0
2X	Alvarenga/MG	ZM369	0
2X	Nova Belém/MG	ZM381	0
2X	Nova Belém/MG	ZM381	0
2X	Nova Belém/MG	ZM381	0
2X	Nova Belém/MG	ZM381	0
2X	Nova Belém/MG	ZM381	0
2X	Nova Belém/MG	ZM381	0
2X	Nova Belém/MG	ZM383	1
2X	Nova Belém/MG	ZM383	1
2X	Nova Belém/MG	ZM406	0
2X	Guiné/BA	ZM407	1
2X	Guiné/BA	ZM407	1
2X	Guiné/BA	ZM407	1
2X	Guiné/BA	ZM407	1

2X	Guiné/BA	ZM407	1
2X	Guiné/BA	ZM407	1
2X	Guiné/BA	ZM407	1
2X	Guiné/BA	ZM407	1
2X	Caraí/MG	ZM582	0
2X	Caraí/MG	ZM583	0
2X	Caraí/MG	ZM584	0
2X	Caraí/MG	ZM585	1
2X	Caraí/MG	ZM587	0
2X	Caraí/MG	ZM588	0
2X	Caraí/MG	ZM589	0
2X	Caraí/MG	ZM590	0
2X	Caraí/MG	ZM591	0
2X	Caraí/MG	ZM592	1
2X	Nova Belém/MG	ZM614	0
2X	Nova Belém/MG	ZM614	0
2X	Nova Belém/MG	ZM614	1
2X	Nova Belém/MG	ZM614	1
2X	Itaipé/MG	ZM655	1
3X	Santana do Riacho/MG	ZM115	0
3X	Santana do Riacho/MG	ZM115	0
3X	Santana do Riacho/MG	ZM115	0
3X	Santana do Riacho/MG	ZM115	1
3X	Santana do Riacho/MG	ZM115	1
3X	Santana do Riacho/MG	ZM116	1
3X	Santana do Riacho/MG	ZM116	1
3X	Santana do Riacho/MG	ZM116	1
3X	Santana do Riacho/MG	ZM116	1
3X	Santana do Riacho/MG	ZM116	1
3X	Santana do Riacho/MG	ZM116	1
3X	Santana do Riacho/MG	ZM116	1
3X	Santana do Riacho/MG	ZM116	1
3X	Santana do Riacho/MG	ZM117	0
3X	Santana do Riacho/MG	ZM117	1
3X	Santana do Riacho/MG	ZM117	1
3X	Santana do Riacho/MG	ZM123	0
3X	Santana do Riacho/MG	ZM123	0
3X	Santana do Riacho/MG	ZM123	0
3X	Santana do Riacho/MG	ZM123	0
3X	Santana do Riacho/MG	ZM123	0
3X	Santana do Riacho/MG	ZM123	0
3X	Santana do Riacho/MG	ZM123	0
3X	Santo Antônio do Itambé/MG	ZM201	1
3X	Santo Antônio do Itambé/MG	ZM201	1
3X	Santo Antônio do Itambé/MG	ZM201	1
3X	Santo Antônio do Itambé/MG	ZM201	1
4X	Cunha/SP	CEPN2	0
4X	Cunha/SP	CEPN2	0
4X	Cunha/SP	CEPN2	1
4X	Cunha/SP	CEPN2	1

4X	Cunha/SP	CEPN3	1
4X	Cunha/SP	CEPN4	1
4X	Santana do Riacho/MG	CIPO	0
4X	Nova Friburgo/RJ	TM23103	1
4X	Nova Friburgo/RJ	TM23103	1
4X	Nova Friburgo/RJ	TM23103	1
4X	Nova Friburgo/RJ	TM23103	1
4X	Nova Friburgo/RJ	TM23103	1
4X	Nova Friburgo/RJ	TM23104	1
4X	Nova Friburgo/RJ	TM23104	1
4X	Nova Friburgo/RJ	TM23105	1
4X	Nova Friburgo/RJ	TM23105	1
4X	Nova Friburgo/RJ	TM23108	0
4X	Biritiba Mirim/SP	ZM006	1
4X	Cotia/SP	ZM035	0
4X	São José do Barriero/SP	ZM041	0
4X	São José do Barriero/SP	ZM041	0
4X	São José do Barriero/SP	ZM041	1
4X	São José do Barriero/SP	ZM049	0
4X	São José do Barriero/SP	ZM049	0
4X	São José do Barriero/SP	ZM049	0
4X	São José do Barriero/SP	ZM049	0
4X	São José do Barriero/SP	ZM049	1
4X	São José do Barriero/SP	ZM053	1
4X	São José do Barriero/SP	ZM053	1
4X	São José do Barriero/SP	ZM053	1
4X	São José do Barriero/SP	ZM059	1
4X	São José do Barriero/SP	ZM059	1
4X	São José do Barriero/SP	ZM059	1
4X	São José do Barriero/SP	ZM059	1
4X	São José do Barriero/SP	ZM061	0
4X	São José do Barriero/SP	ZM061	1
4X	São José do Barriero/SP	ZM063	1
4X	São José do Barriero/SP	ZM063	1
4X	São José do Barriero/SP	ZM064	1
4X	Santana do Riacho/MG	ZM076	0
4X	Santana do Riacho/MG	ZM076	1
4X	Santana do Riacho/MG	ZM076	1
4X	Santana do Riacho/MG	ZM076	1
4X	Santana do Riacho/MG	ZM076	1
4X	Piquete/SP	ZM099	1
4X	Piquete/SP	ZM105	0
4X	Piquete/SP	ZM105	0
4X	Piquete/SP	ZM105	0
4X	Piquete/SP	ZM105	0
4X	Piquete/SP	ZM112	0
4X	Biritiba Mirim/SP	ZM119	1
4X	Biritiba Mirim/SP	ZM119	1
4X	Biritiba Mirim/SP	ZM121	1
4X	Biritiba Mirim/SP	ZM121	1

4X	Santana do Riacho/MG	ZM125	0
4X	Santana do Riacho/MG	ZM125	0
4X	Santana do Riacho/MG	ZM125	0
4X	Biritiba Mirim/SP	ZM134	0
4X	Biritiba Mirim/SP	ZM134	0
4X	Biritiba Mirim/SP	ZM134	0
4X	Biritiba Mirim/SP	ZM134	0
4X	Biritiba Mirim/SP	ZM134	1
4X	Biritiba Mirim/SP	ZM134	1
4X	Biritiba Mirim/SP	ZM134	1
4X	Biritiba Mirim/SP	ZM134	1
4X	Biritiba Mirim/SP	ZM134	1
4X	Biritiba Mirim/SP	ZM134	1
4X	Biritiba Mirim/SP	ZM137	1
4X	Biritiba Mirim/SP	ZM137	1
4X	Biritiba Mirim/SP	ZM138	1
4X	Biritiba Mirim/SP	ZM145	0
4X	Biritiba Mirim/SP	ZM145	1
4X	Biritiba Mirim/SP	ZM145	1
4X	Biritiba Mirim/SP	ZM145	1
4X	Biritiba Mirim/SP	ZM145	1
4X	Biritiba Mirim/SP	ZM145	1
4X	Biritiba Mirim/SP	ZM145	1
4X	Biritiba Mirim/SP	ZM147	0
4X	Biritiba Mirim/SP	ZM147	1
4X	Biritiba Mirim/SP	ZM147	1
4X	Biritiba Mirim/SP	ZM147	1
4X	Biritiba Mirim/SP	ZM147	1
4X	Biritiba Mirim/SP	ZM147	1
4X	Biritiba Mirim/SP	ZM147	1
4X	Biritiba Mirim/SP	ZM148	1
4X	Biritiba Mirim/SP	ZM148	1
4X	Biritiba Mirim/SP	ZM148	1
4X	Biritiba Mirim/SP	ZM148	1
4X	Biritiba Mirim/SP	ZM150	1
4X	São José do Barriero/SP	ZM158	0
4X	São José do Barriero/SP	ZM158	0
4X	São José do Barriero/SP	ZM158	0
4X	São José do Barriero/SP	ZM158	1
4X	São José do Barriero/SP	ZM159	0
4X	São José do Barriero/SP	ZM159	0
4X	São José do Barriero/SP	ZM159	1
4X	Passa Quatro/MG	ZM164	0
4X	Passa Quatro/MG	ZM169	1
4X	Santo Antônio do Itambé/MG	ZM185	0
4X	Santo Antônio do Itambé/MG	ZM185	0
4X	Santo Antônio do Itambé/MG	ZM185	0
4X	Santo Antônio do Itambé/MG	ZM185	0
4X	Santo Antônio do Itambé/MG	ZM185	0
4X	Ouro Preto/MG	ZM242	1

4X	Ouro Preto/MG	ZM242	1
4X	Ouro Preto/MG	ZM242	1
4X	Ouro Preto/MG	ZM245	0
4X	Ouro Preto/MG	ZM245	1
4X	Ouro Preto/MG	ZM245	1
4X	Ouro Preto/MG	ZM245	1
4X	Ouro Preto/MG	ZM245	1
4X	Ouro Preto/MG	ZM254	1
4X	Ouro Preto/MG	ZM254	1
4X	Alvarenga/MG	ZM370	1
4X	Paty do Alferes/RJ	ZM461	1
4X	Paty do Alferes/RJ	ZM461	1
4X	Paty do Alferes/RJ	ZM466	1
4X	Paty do Alferes/RJ	ZM466	1
4X	Paty do Alferes/RJ	ZM466	1
4X	Paty do Alferes/RJ	ZM466	1
4X	Paty do Alferes/RJ	ZM466	1
4X	Paty do Alferes/RJ	ZM466	1
4X	Paty do Alferes/RJ	ZM466	1
4X	Paty do Alferes/RJ	ZM466	1
4X	Paty do Alferes/RJ	ZM466	1
4X	Santa Maria Madalena/RJ	ZM490	1
4X	unknown	ZM694	0
4X	unknown	ZM694	1
4X	unknown	ZM695	0
4X	unknown	ZM695	1

Table S2. Chi-square analyses test for residuals to compare the impact of ploidy on fruit set for each cytotype of *Zygopetalum mackayi*. Diploid, 2n = 48 (2*x*); triploid, 2n = 72 (3*x*); and tetraploid, 2n = 96 (4*x*). Significance level of 0.05.

**Ploidy**	**Values**	**Fruits developed to completion**	**Fruit abortions**	**Total**
2*x*	Count	22	42	64
	Expected Count	36.8	27.2	64.0
	Adjusted Residuals	-4.4	4.4	
3*x*	Count	16	11	27
	Expected Count	15.5	11.5	27.0
	Adjusted Residuals	0.2	-0.2	
4*x*	Count	89	41	130
	Expected Count	74.7	55.3	130.0
	Adjusted Residuals	4.0	-4.0	
Total	Count		94	221
	Expected Count		94.0	221.0

Chi-Square Test

	**Value**	**df**	**Asymptotic Significance (2-sided)**
Pearson Chi-Square	20.427[a]	2	<0.001
Likelihood Ratio	20.491	2	<0.001
N of Valid Cases	221		

a. 0 cells (0.0%) have expected Count less than 5. The minimum expected count is 11.5.
